# Interpretable machine learning for early neurological deterioration prediction in atrial fibrillation-related stroke

**DOI:** 10.1038/s41598-021-99920-7

**Published:** 2021-10-18

**Authors:** Seong-Hwan Kim, Eun-Tae Jeon, Sungwook Yu, Kyungmi Oh, Chi Kyung Kim, Tae-Jin Song, Yong-Jae Kim, Sung Hyuk Heo, Kwang-Yeol Park, Jeong-Min Kim, Jong-Ho Park, Jay Chol Choi, Man-Seok Park, Joon-Tae Kim, Kang-Ho Choi, Yang Ha Hwang, Bum Joon Kim, Jong-Won Chung, Oh Young Bang, Gyeongmoon Kim, Woo-Keun Seo, Jin-Man Jung

**Affiliations:** 1 Department of Neurology, Korea University Ansan Hospital, Korea University College of Medicine, Gojan 1-Dong, Danwon-Gu, Ansan-Si, Gyeonggi-Do 15355 South Korea; 2 Department of Neurology, Korea University Anam Hospital, Korea University College of Medicine, Seoul, South Korea; 3 Department of Neurology, Korea University Guro Hospital, Korea University College of Medicine, Seoul, South Korea; 4 Department of Neurology, Seoul Hospital, Ewha University College of Medicine, Seoul, South Korea; 5 Department of Neurology, Eunpyeong St. Mary’s Hospital, The Catholic University of Korea, Seoul, Korea; 6 Department of Neurology, Kyung Hee University College of Medicine, Seoul, South Korea; 7 Department of Neurology, Chung-Ang University College of Medicine, Chung-Ang University Hospital, Seoul, South Korea; 8 Department of Neurology, Hanyang University Myongji Hospital Seoul, Seoul, South Korea; 9 Department of Neurology, Jeju National University, Jeju, South Korea; 10 Department of Neurology, Chonnam National University Hospital, Chonnam, South Korea; 11 Department of Neurology, Chonnam National University Hwasun Hospital, Hwasun, South Korea; 12 Department of Neurology, Kyungpook National University Hospital, Dae-gu, South Korea; 13 Department of Neurology, Asan Medical Center, University of Ulsan College of Medicine, Seoul, South Korea; 14 Department of Neurology and Stroke Center, Samsung Medical Center, 81, Irwon-Ro, Gangnam-Gu, Seoul, 06351 South Korea; 15 Korea University Zebrafish Translational Medical Research Center, Ansan, South Korea

**Keywords:** Neuroscience, Neurology

## Abstract

We aimed to develop a novel prediction model for early neurological deterioration (END) based on an interpretable machine learning (ML) algorithm for atrial fibrillation (AF)-related stroke and to evaluate the prediction accuracy and feature importance of ML models. Data from multicenter prospective stroke registries in South Korea were collected. After stepwise data preprocessing, we utilized logistic regression, support vector machine, extreme gradient boosting, light gradient boosting machine (LightGBM), and multilayer perceptron models. We used the Shapley additive explanation (SHAP) method to evaluate feature importance. Of the 3,213 stroke patients, the 2,363 who had arrived at the hospital within 24 h of symptom onset and had available information regarding END were included. Of these, 318 (13.5%) had END. The LightGBM model showed the highest area under the receiver operating characteristic curve (0.772; 95% confidence interval, 0.715–0.829). The feature importance analysis revealed that fasting glucose level and the National Institute of Health Stroke Scale score were the most influential factors. Among ML algorithms, the LightGBM model was particularly useful for predicting END, as it revealed new and diverse predictors. Additionally, the effects of the features on the predictive power of the model were individualized using the SHAP method.

## Introduction

Early neurological deterioration (END) is a sudden worsening of neurological symptoms during the acute period of stroke. END leads to devastating clinical outcomes despite marked advances in acute stroke management over the past several years. The incidence of END is considerably high, ranging from 5 to 40%, and is associated with a poor 3-month clinical prognosis and high mortality^1,2^. The standard treatment strategy for END has not been established, and an accurate prediction of END is unavailable in clinical practice owing to its complexity and heterogeneity. In addition, there has been no consensus on the definition. Therefore, various inclusion criteria and study designs have been used, with some studies preferring to define END according to specific stroke subtypes (e.g., cardioembolism), making each predictor and recent nomograms difficult to use in real-world clinical practice^3–6^. Those obstacles make it difficult to design a prospective early detection and early interventional study. Accurate prediction of END is of paramount importance not only for the prognostication but also to motivate prospective, early interventional studies to prevent or restore END in patients with stroke.

Of the etiologies attributed to cardioembolic stroke, atrial fibrillation (AF) is one of the predictors of END^7,8^. Several markers, including clinical, radiological, and laboratory findings, have been associated with END in AF-related stroke^9–11^. However, in those studies, using a single marker had limited predictive power, since the diverse biomarkers and imaging markers relevant to END in AF-related stroke were not considered at the same time.

Continuous advancements in machine learning (ML) algorithms have led to their wide application in the medical field, as numerous variables and massive data can be included and analyzed. In contrast to transitional statistical models, ML models are compatible with predicting complex clinical events that can be affected by diverse situations and conditions. Nevertheless, the clinical application of ML models has been limited owing to the ‘black box problem’ of interpretability and explanation^12^. Therefore, it is essential that ML models be interpretable to the current medical fields^13^. The Shapley additive explanations (SHAP) method is a novel, cutting-edge method designed to aid in clinical interpretation and intuitive understanding of feature importance by providing visualizations of the relationship between each feature and the associated predictive power^14^. Therefore, the aim of our study was to develop an interpretable ML model that could predict END using the feature importance technique in AF-related stroke using a real-world multicenter cohort database.

## Methods

### Study design and participants

The dataset from this study can be provided by the corresponding author upon reasonable request.

This study was based on the Korean Atrial Fibrillation Evaluation Registry in Ischemic Stroke Patients (K-ATTENTION), a real-world cohort composed of prospective stroke registries from 11 tertiary centers in South Korea. K-ATTENTION focused on characteristics, oral anticoagulant use, and outcomes in AF-related stroke patients^15^. Between January 2013 and December 2015, patients who were admitted to one of the participating centers within 7 days of stroke onset were enrolled. Detailed information regarding management and follow-up of the included patients has been provided previously^15^. In our study, only those who arrived at the hospital within 24 h of symptom onset and had information regarding END were included. Using the internet-based clinical recording system, we acquired the following patient information from each center: demographic characteristics, vascular risk factors, brain imaging results, laboratory findings, pre-admission medication histories, stroke severity on admission (according to the National Institutes of Health Stroke Scale [NIHSS] score), and functional status (modified Rankin score [mRS]). Additional information on variable acquisition and evaluation is provided in Supplemental Table I and Supplemental Methods I. This study followed the Transparent Reporting of a multivariable prediction model for Individual Prognosis or Diagnosis (TRIPOD) reporting guidelines^16^. The institutional review boards of Korea University Ansan Hospital (2016AS0051), Korea university Anam hospital, Korea University Guro Hospital, Ewha University College of Medicine, Eunpyeong St. Mary’s Hospital, Kyung Hee University College of Medicine, Chung-Ang University Hospital, Hanyang University Myongji Hospital, Jeju National University, Chonnam National University Hospital, Chonnam National University Hwasun Hospital, Kyungpook National University Hospital, Asan Medical Center, and Samsung Medical Center approved the study. The need for informed consent was waived by the ethics committee of all participating centers due to the retrospective design of the study using anonymous and de-identified information.

### Definition of END as the main outcome

END was defined as an increase of at least 2 points in the total NIHSS score and at least 1 point on the level of consciousness or motor item score within 72 h of arrival at the hospital^17^.

### Data splitting and preprocessing

Binary variables with less than 80% missing values and multinomial and numeric variables with less than 60% missing values were included to generate the available dataset^18^. In the first step, 25% of the dataset was randomly separated according to END stratification and used only in the final evaluation of model performance as a test set. The remaining 75% of the dataset was used as a training set for hyperparameter determination and training processes using leave-one-out cross-validation. In this data splitting process, we used the stratified random sampling method, stratifying institution sites to reduce the multi-site correction problem. Isolation forest and multivariate imputation by chained equations were used for outlier detection and imputation. Details of the methods are provided in Supplemental Methods II.

### Feature selection and feature importance analysis

Recursive feature elimination^19^ was used to select the top-k ranked features that contributed to the overall model performance of the area under the receiver operating characteristic curve (AUROC). In this feature selection analysis, we also included institution site variable to evaluate the multi-site correction issue. Since the purpose of this study was to evaluate a predictive model based on variables that can be obtained at the time of admission to the hospital, variables that cannot be evaluated at the initial time point were excluded. To measure and rank the contribution of each variable, we obtained mean absolute SHAP values^14^ with a gradient boosted tree-based model, light gradient boosting machine (LightGBM)^20^, which can deal natively with categorical features^21^ using leave-one-out cross-validation. The positive SHAP value for each variable indicated that the variable contributed positively to the model’s positive prediction, and vice versa. We performed an additional stepwise process to prevent underestimation of the relative importance of features due to multicollinearity, the details of which are described in Supplemental Methods III.

### Modeling

We selected and tested one conventional statistical model, logistic regression^22^, as a baseline comparator, and four popular ML models: support vector machine^23^, extreme gradient boosting^24^ (XGBoost), Light GBM, and multilayer perceptron^25^ (MLP) with a basic architecture. Detailed instructions for the applied models are provided in Supplemental Methods IV. All the processes were implemented in Python 3.8.2 with TensorFlow-GPU 2.4.0^26^ and scikit-learn 0.22.1^27^ libraries.

### Primary outcome and evaluation criteria

AUROC was chosen as a primary evaluation metric for model performance, and all cross-validation and early stopping strategies in the modeling process were performed to maximize the AUROC score. The models were evaluated for the frequency of confident answers and errors, with a threshold of 0.50.

### Statistical analysis

Categorical variables are presented as number (percentage), and continuous variables are presented as mean ± standard deviation or median (interquartile range), as appropriate. A simple comparison was performed using the *χ*^2^ test for categorical variables and the Kruskal–Wallis test for continuous variables. Data analyses were performed using IBM SPSS version 20 software (IBM Corp. Armonk, NY, USA). The AUROC, with a 95% confidence interval (CI), was calculated using the Delong method and a CI that spanned 0.50 or more was not considered statistically different from a random performance^28^. To evaluate the calibration error of the models, the Brier score, which is the mean squared difference between the predicted probability and the actual outcome, was calculated^29^, with a lower score indicating better probabilistic prediction accuracy. In addition, the area under the precision-recall curve, accuracy, precision, recall, and F1 score were calculated as secondary outcome metrics. We also calculated the sensitivity, specificity, and precision values for various thresholds. The significance level was set at *p*< 0.05 and Bonferroni correction was used for multiple comparisons of the AUROC between models.

## Results

### Comparisons of baseline characteristics

**Figure 1:**
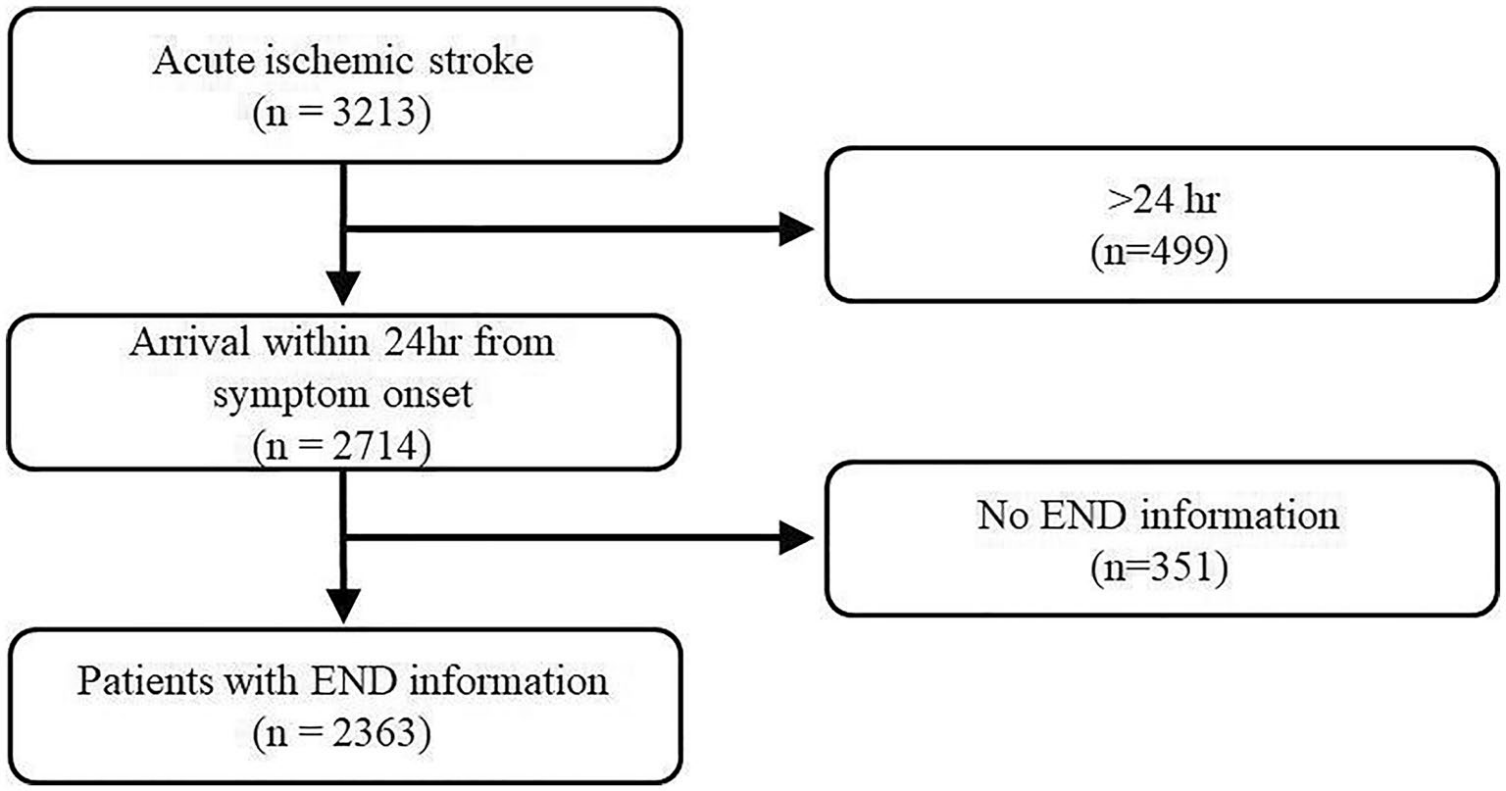
Flowchart of included patients.

Figure 1 shows the patient flow chart. A total of 2,363 patients were included in this study, of whom 318 (13.5%) had END. Comparisons of baseline clinical characteristics and MRI variables are listed in Supplemental Tables II and III.

### Missing value imputation

The binary variables with missing values over 80% and multinomial and numeric variables with missing values over 60% were excluded from the model construction dataset according to the missing data imputation strategy described in a previous study^30^. The variables were as follows: all Holter monitoring parameters, smoking pack-years, alcohol consumption, duration of PR and P-axis wave on electrocardiogram, susceptibility vessel sign (SVS) size, urine albumin, serum free fatty acid level, brain natriuretic peptide (BNP), N-terminal pro-BNP, and troponin T. The remaining missing values were imputed, with non-categorical missing values imputed using the multivariate imputation by chained equations imputation method, and categorical missing values were replaced with a single constant of -1. Details concerning the number of missing values for each variable are listed in Supplemental Table IV.

### Model performances

**Figure 2:**
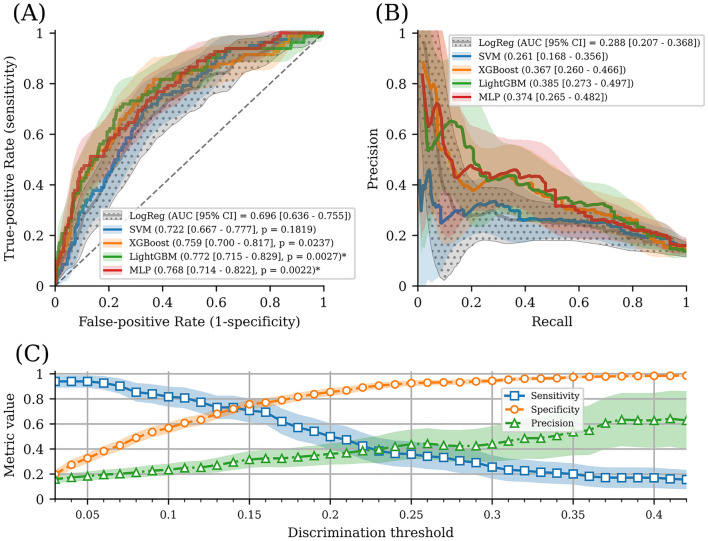
Model performance. (**A**)**,** Solid lines and shades represent receiver operating characteristics curves and its 95% confidence intervals. An asterisk (*) indicates significant difference (*P* < 0.005) in comparison with logistic regression. (**B**)**,** Solid lines and shades represent precision-recall curves and its 95% confidence intervals. Only the confidence intervals of the baseline model (logistic regression, “LogReg”) are represented with polka dot pattern in both plots. (**C**)**,** Detailed performance analysis for the best model (LightGBM) in different discrimination thresholds. Solid lines and shades represent mean values and 95% confidence intervals in each variable. Abbreviations: AUC, area under the curve; CI, confidence interval; LogReg, Logistic regression; SVM, Support vector machine; XGBoost, Extreme gradient boosting; LightGBM, light gradient boosting machine; MLP, Multilayer perceptron.

A flow diagram of the ML model development process is presented in Supplemental Figure I. The performance of each model is shown in Table 1, and the receiver operating characteristic curve and precision-recall curve are shown in Fig. 2. LightGBM had the highest AUROC value (0.772 [0.715–0.829]); however, there was no significant difference between the ML models. Light GBM and MLP had significantly higher AUROC values than logistic regression (p = 0.003 and 0.002, respectively). At various discrimination thresholds, the sensitivity, specificity, and precision of the model were calculated, and our model showed relatively superior performance for specificity.

**Table 1:** Comparison of model performance.

Model	AUROC [95% CI]	AUPRC [95% CI]	Brier score	ACC (%)	Precision	Recall	F1 score	p value†
**Baseline model**								
Logistic regression	0.696 [0.636–0.755]	0.288 [0.207–0.368]	0.110	86.5	0.253	0.585	0.353	
**Machine learning models**								
SVM	0.722 [0.667–0.777]	0.261[0.168–0.356]	0.112	86.2	0.254	0.695	0.373	0.182
XGBoost	0.759 [0.700–0.817]	0.367 [0.260–0.466]	0.105	86.5	0.349	0.537	0.423	0.024
LightGBM	0.772 [0.715–0.829]	0.385 [0.273–0.497]	0.103	86.7	0.328	0.695	0.445	0.003*
MLP	0.768 [0.714–0.822]	0.374 [0.265–0.482]	0.103	86.9	0.432	0.463	0.447	0.002*

*Significant difference at *p* < 0.005.

^†^Comparison with logistic regression on AUROC.

Abbreviations: AUROC, area under the receiver operating characteristic curve; AUPRC, area under the precision-recall curve; SVM, support vector machine; XGBoost, extreme gradient boosting; LightGBM, light gradient boosting machine; MLP, multilayer perceptron.

### Identification of important features

**Figure 3:**
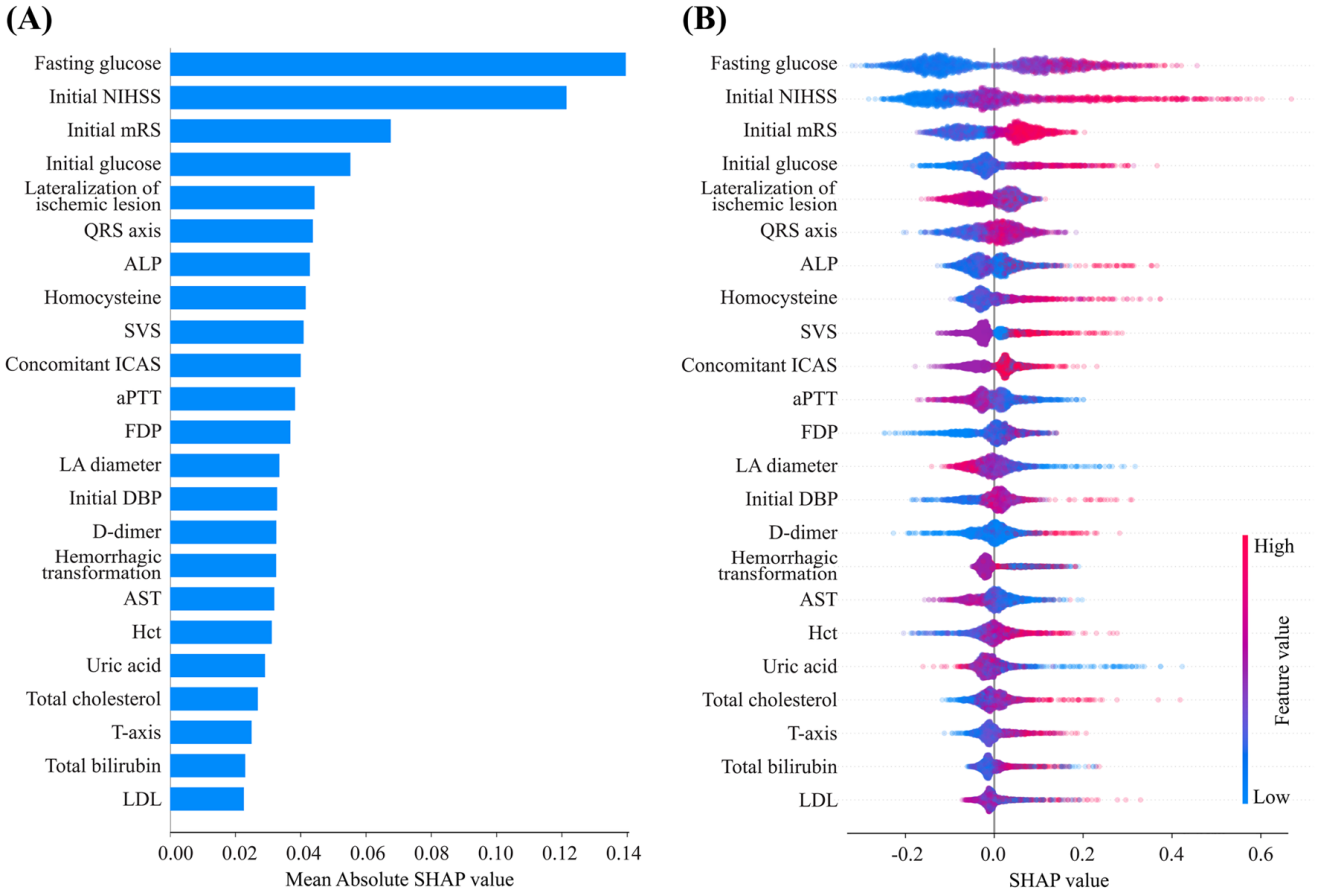
Matrix plots of top 23 important features. Bar plot (**A**) and violin plot (**B**). In the bar plot, the SHAP value implies the degree of contribution of a specific feature. The higher the SHAP value, the larger the model contribution of a specific feature. In the violin plot, each dot represents one patient and accumulates vertically to depict the density. The color reflects the high and low values of each feature, with the red color indicating a higher value and the blue color indicating a lower value. The X-axis of the graph represents the SHAP value, and a positive SHAP value indicates that it contributes positively to predicting the model, and that the probability of END occurring is high, and vice versa. Abbreviations: NIHSS, National Institute of Health Stroke Scale; mRS, modified Rankin scale; ALP, alkaline phosphatase; SVS, susceptibility vessel sign; ICAS, intracranial atherosclerosis; aPTT, activated partial thromboplastin time; FDP, fibrin degradation product; LA, left atrium; DBP, diastolic blood pressure; AST, aspartate aminotransferase; Hct, hematocrit; LDL, low-density lipoprotein.

From the recursive feature elimination, a total of 23 features were selected as important features. The SHAP feature importance matrix plots show important features according to the degree of contribution (bar plot, Fig. 3A) and the overall correlation and directionality between features and the SHAP value (violin plot, Fig. 3B) during model construction. Among them, fasting glucose levels and initial NIHSS score contributed the most to the model. The next highest-ranking features were the initial mRS and initial glucose level. All other features contributed less to the model. It was confirmed that the institute site variable had not significantly affected the model. In addition, most of the continuous variables, such as fasting glucose, initial NIHSS score, initial mRS, initial glucose, QRS axis, alkaline phosphatase, homocysteine, fibrin degradation product, initial diastolic blood pressure, D-dimer, hematocrit, total cholesterol, and T axis tended to be positively correlated with END. Activated partial thromboplastin time, aspartate aminotransferase, total bilirubin, and low-density lipoprotein (LDL) cholesterol showed complex patterns with mixed positive and negative trends. LA diameter and uric acid levels showed a negative correlation.

**Figure 4:**
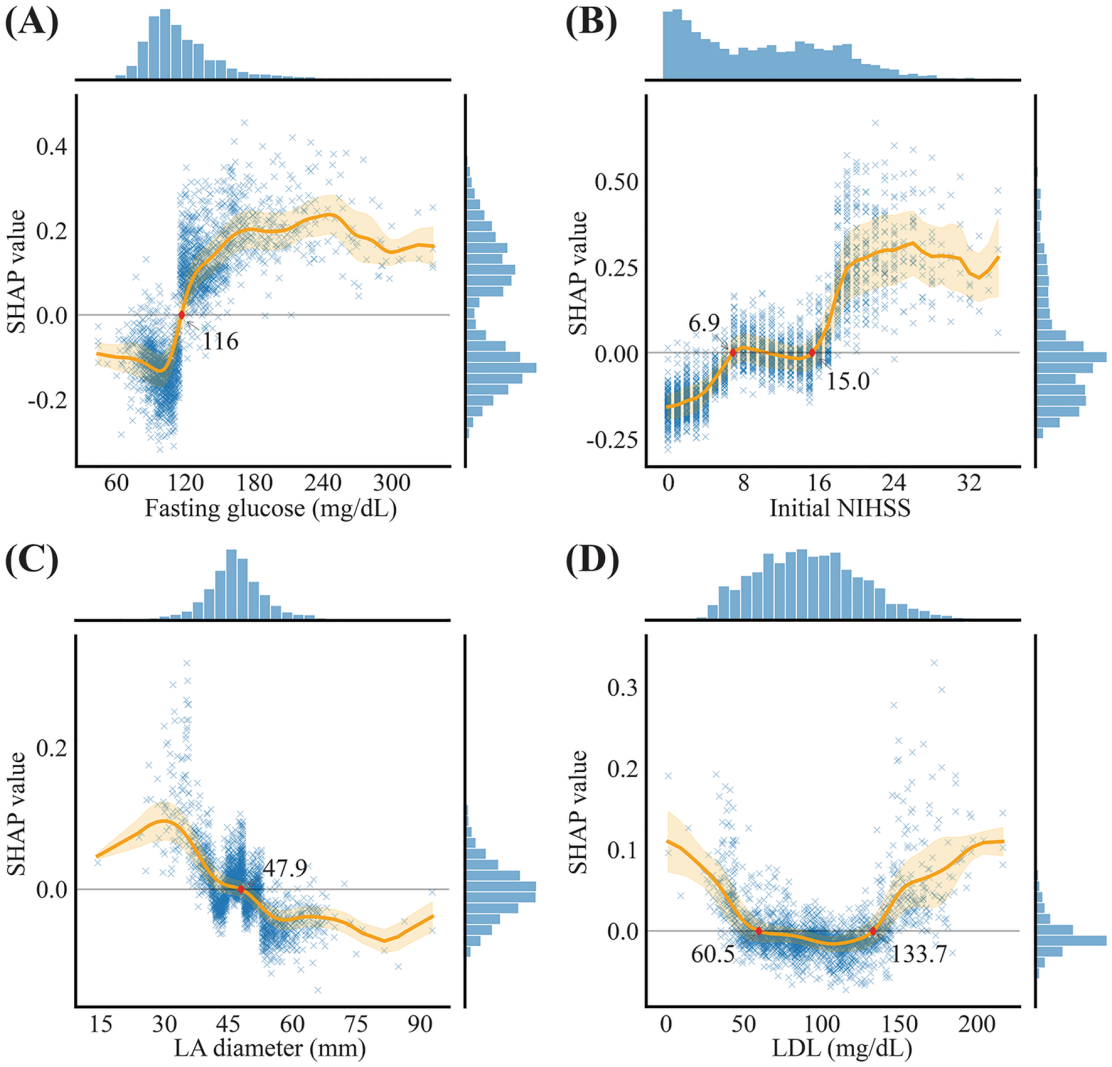
Partial SHAP dependence plot of the four representative features. Values are plotted with a scatter plot and a regression line represented with the orange line of mean and shade of SD. A red diamond represents a cut-off value of the variable. Histograms on the right and top of each plot are distributions of the SHAP and values of variables. Abbreviations: NIHSS, National Institute of Health Stroke Scale; LA, left atrium; LDL, low- density lipoprotein.

SHAP values corresponding to changes in the four representative features are presented in partial SHAP dependence plots (Fig. 4), and other representative feature plots are listed in Supplemental Figure II. The fasting glucose level and initial NIHSS score showed a positive correlation with the sigmoid or double sigmoid curve. The LA diameter declined negatively. LDL cholesterol was associated with a U-shaped trend line that initially showed a declining tendency followed by a reversed, increasing trend. The cut-off value for each variable that could predict the positive and/or negative probability of END occurrence is marked on each graph.

Lateralization of ischemic lesions, concomitant intracranial atherosclerosis, SVS signs, and hemorrhagic transformation were included as categorical variables. The presence of concomitant intracranial atherosclerosis, SVS signs, and symptomatic ICH among hemorrhagic transformations is likely to related to END occurrence. Posterior circulation lesions were unlikely to develop END (Supplemental Figure II).

In addition, we acquired information about the importance and contribution of each patient according to the specific features selected during modeling. Representative cases are summarized in Supplemental Figure III.

## Discussion

In this study, we first demonstrated that integrated ML algorithms can be applied to predict END in AF-related stroke cases. Among the ML models investigated, LightGBM had the best performance, with an AUROC value of 0.772. This is a novel method with efficient computational power and wide scalability for processing categorical, multidimensional, and incredibly large datasets^20^, which makes it a suitable ML model in clinical settings. In addition, this model was implemented using SHAP, which can visualize the level of contribution and directionality of specific input features using the entire dataset as well as individual patient information.

The highest contributing feature in our study was the fasting glucose level, followed by the initial NIHSS score. These variables have been consistently reported as risk factors for END in all-type as well as AF-related stroke cases^2,10,11^. A possible explanation is that the impairment of glucose control causes vascular endothelial dysfunction^31^, post-ischemic inflammatory response, and neuroprotective heat-shock chaperone gene attenuation^32^, which could exacerbate post-stroke brain damage through increasing lactate production and leading to the breakdown of the blood–brain barrier, development of brain edema and hemorrhagic transformation, and enlargement of infarct volume^18^. The initial neurological functional deficits represented using the NIHSS score and mRS were also known to be prone with symptomatic intracranial hemorrhage, malignant edema or stroke-related infection^33^, which are important causes of END^2^. In fact, symptomatic cerebral hemorrhage of the hemorrhagic transformation subtype was positively associated with END in this study. In addition, homocysteine, which is related to vascular endothelial dysfunction^3^, and fibrin degradation product and D-dimer, which are important hematologic markers related to the coagulation system and thrombosis, were important features similar to previous studies^34–36^. Other features were SVS presence implying large-size infarction; specific ischemic lesion location limited to anterior or posterior circulation^37^; cardiac electrophysiological, and echocardiographic markers such as QRS axis, T axis and left atrium diameter; alkaline phosphatase^38,39^ as surrogate markers of atherosclerosis, systemic inflammation, malnutrition, or metabolic syndrome; and the burden of atherosclerosis, such as concurrent intracranial atherosclerosis^37,40^. Among cholesterol lipoproteins, total cholesterol and LDL were included as important features in this study, which have been previously reported as important predictors^41^.

Interestingly, the clinical implication of cut-off values in selected features may be applicable to real-world clinical practice. With regard to initial stroke severity measured using the NIHSS, cut-off values in the SHAP partial dependence plot were presented according to the effect direction of END prediction, suggesting that patients with severe stroke (NIHSS ≥ 15) tended to develop END, thus emphasizing that awareness and close medical attention are necessary for these patients, and patients with mild to moderate stroke (NIHSS ≤ 6) have a lower chance of developing END. Some cut-off values were statistically significantly similar to the clinical values. Indeed, the cut-off value for fasting glucose predicting END in our study was 116 mg/dL, which corresponds to the current diagnostic criteria for diabetes mellitus (≥ 126 mg/dL)^42^.

The SHAP and its corresponding graphs, which were used to evaluate the effect of continuous variables on the prediction of END, were characterized by four patterns. First, a positive correlation with or without a sigmoid or double sigmoid shape was observed. The initial glucose level, fasting glucose level, initial NIHSS score, homocysteine, D-dimer, fibrin degradation product, initial diastolic blood pressure, total cholesterol, QRS-axis, and T-axis corresponded to this pattern. Most of these variables have been reported as predictors of END in previous studies^2^. Second, a U-shaped or J-shaped pattern with both cut-off values was observed for aspartate aminotransferase, alkaline phosphatase, total bilirubin, and LDL cholesterol. The lower cut-off value of each feature may have been associated with poor nutritional status and over the upper cut-off value may imply comorbid conditions including liver disease and hyperlipidemia. However, it is not possible to investigate the underlying pathomechanisms of these phenomena in this study. Third, the following had a negative correlation with END, with a reverse S or J shape: LA diameter and uric acid. In particular, the negative association between LA diameter and END is not consistent with the positive correlation found in a previous report^43^. However, more accurate parameters, such as the LA volume index, have recently been identified as important predictors. Considerable imputation (21.1%) could lead to incorrect directions and biased results. Finally, a bizarre pattern with multidirectionality was observed in the activated partial thromboplastin time.

One strength of our study is that our interpretable ML model was constructed using many variables, including demographics and laboratory, radiological, and echocardiographic findings, all of which can be obtained upon arrival at the hospital. Additionally, an interpretable and explainable ML model was created to promote the use of applications for making clinical decisions. Our study demonstrates the potential of interpretable ML methods to predict END and individualize such predictions. Previous studies have focused on each risk factor individually and its pathophysiological interpretation, but there has been a shortage of clinical use of a large combination of variables once^3–5^. Moreover, no standardized risk stratification scheme for predicting END has been available until now. Therefore, our ML model has the advantage of being able to predict END using diverse variables extracted from real-world clinical situations upon arrival at the hospital.

Our study has some limitations. First, the implementation and evaluation of the model were difficult to generalize because of the lack of external validation. Although this study is based on a multicenter dataset, it is difficult to clearly evaluate the exportability of the model if external validation is not carried out, particularly considering that Light GBM is prone to an overfitting problem. However, to the best of our knowledge, this is the first ML study based on a multicenter and nationwide dataset reflecting various environments across centers. This could partially contribute to the generalizability and representativeness of our ML model because our model could be generally applicable in various external conditions. Nonetheless, further verification is required through well-designed prospective clinical studies and external validation in the future. Second, since this was a registry-based study with a retrospective design, the ML model’s performance is not sufficient to be an absolute criterion for clinical use. It is necessary to develop a more accurate prediction model, and discover novel biomarkers, especially using neuroimaging with more advanced analysis methodology, for a deeper understanding of the pathophysiology, in parallel. Third, a considerable amount of data was missing because of the multicenter retrospective nature of the study. Although imputation of missing data was performed using the ML technique, the results may be biased and contradict previous findings. In particular, it seemed to occur with some elements (such as left atrial size) that were less important. In addition, laboratory and imaging protocols in each center were not concretely established before data collection. Additionally, Holter and electrocardiography parameters were not standardized; therefore, many variables were excluded.

In conclusion, ML algorithms, using the LightGBM model in particular, can be used to predict END in AF-related stroke cases. New and diverse predictors for END were revealed through this ML model, suggesting that the pathophysiology of END development could be a complex mechanism. Further verification through prospective clinical studies is required.

## Supplementary Information

Supplementary Information.
