# Supplementary material for: Interpretable machine learning for early neurological deterioration prediction in atrial fibrillation-related stroke
Source: Sci Rep. 2021 Oct 18;11:20610. doi: 10.1038/s41598-021-99920-7 (PMC8523653; doi:10.1038/s41598-021-99920-7)
Supplement: Supplementary file 1 — Supplementary Information. [file 41598_2021_99920_MOESM1_ESM.pdf]

Supplemental Material

Interpretable Machine Learning for Early Neurological Deterioration Prediction in Atrial Fibrillation-Related Stroke

Supplemental Methods

I. Evaluation and acquisition of feature variables used in the model

II. Outlier detection and imputation methods

III. Preventing underestimation of feature importance

IV. Applied model instructions

Supplemental Tables

I. Evaluation and acquisition of variables used in the model

II. Comparison of baseline clinical characteristics

III. Comparison of MRI variables

IV. Missing values

Supplemental Figures

I. Flowchart of data preprocessing and machine learning model construction

II. SHAP value changes according to features

III. Representative cases with SHAP values

Supplemental Methods

I. Evaluation and acquisition of feature variables used in the model

Vascular risk factors included smoking status, congestive heart failure, hypertension, diabetes mellitus, coronary artery disease, peripheral artery disease, and prior stroke or transient ischemic attack. Brain MRI findings, which were categorized into pre-specified classifications, were acquired. Lateralization features (right anterior *vs*. left anterior *vs*. right posterior *vs*. left posterior *vs*. bilateral or diffuse multifocal circulation) and lesion patterns (single cortico-subcortical *vs*. cortical *vs*. subcortical [> 15 mm] *vs*. subcortical [< 15 mm] *vs*. small scattered lesions in one vascular territory *vs*. confluent and an additional lesion in one vascular territory *vs*. multiple lesions in multiple vascular territories) shown on diffusion-weighted imaging and the presence of susceptible vessel sign on gradient-echo images were recorded. The presence of intracranial and/or extracranial atherosclerosis (stenotic degree ≥ 50% of the total lumen) through MRI or CT angiography was classified as none *vs*. symptomatic or asymptomatic. Hemorrhagic transformation was evaluated by follow-up MRI or CT and firstly classified into none vs hemorrhagic transformation type 1 vs hemorrhagic transformation type 2 vs parenchymal hematoma type 1 vs parenchymal hematoma type 2 vs symptomatic intracranial hemorrhage [1]. Then subdivided into none *vs*. non-significant hemorrhagic transformation (including hemorrhagic infarction type 1, 2, and parenchymal hematoma type 1) vs significant hemorrhagic transformation (including parenchymal hematoma type 2 and symptomatic intracranial hemorrhage) [2]. Laboratory examinations included complete blood count, total cholesterol, triglycerides, high-density lipoprotein cholesterol, low-density lipoprotein cholesterol, C-reactive protein, glucose, D-dimer, troponin I or T, and creatinine. Twelve-lead electrocardiography (ECG) was performed upon admission. The echocardiographic parameters included left ventricular ejection fraction and left atrial diameter. Atrial fibrillation (AF) was documented on ECG during hospitalization and/or 24-hour Holter monitoring and/or continuous ECG monitoring in the stroke unit. Additional information about AF included the time of diagnosis (prior to admission *vs.* during hospitalization *vs.* after discharge) and the type (paroxysmal *vs.* persistent).

II. Outlier detection and imputation methods

Isolation forest [3] was used for outlier detection, and the outliers were replaced with the closest value among non-outlier values in the training set. We performed multivariate imputation chained equations (MICE) [4] for non-categorical variables. The MICE models each variable with missing values as a function of other variables and uses that estimate for imputation. The range of imputation was limited to the minimum and maximum values of the training set. The imputed values for discrete-value variables were rounded to the nearest discrete value, and categorical features were one-hot-encoded.

III. Preventing underestimation of feature importance

Since the relative importance of features can be underestimated for correlated variables [5], we performed hierarchical clustering on the features' Spearman rank-order correlations in every recursion during recursive feature elimination and evaluated every feature with a new feature set, excluding all others in the cluster of features. We repeated the calculation of Spearman's r and kept a feature from each cluster, plotting a dendrogram, and clustering features or clusters twice. After the double clustering of features, it was confirmed that there were no clusters with Ward's linkage < 1.

IV. Applied model instructions

We selected and tested a conventional statistical model, three different popular machine learning models, and one deep learning model: logistic regression [6], Extreme gradient boosting (XGBoost) [7], support vector machine [8], light gradient boosting machine (LightGBM) [9], and a multilayer perceptron (MLP) [10]. Logistic regression is a member of the generalized linear model family, which uses a logistic function to model a binary dependent variable. XGBoost and LightGBM are implementations of gradient-boosted decision trees. Unlike XGBoost, which using level-wise tree growth, LightGBM divides a leaf node with max delta loss in every tree growth. The leaf-wise algorithm can be vulnerable to overfitting but it allows much faster training and lowering more loss. Support vector machine constructs a hyperplane in a high-dimensional space, allowing for the classification of tasks. The MLP has a basic architecture of fully connected neural network, where all the nodes in one layer are connected to the neurons in the next layer. During the MLP training, early stopping strategy, batch normalization [11], and dropout [12] techniques were applied to prevent overfitting. The Glorot uniform initializer [13], which is used for initializing the activation function, and the Nesterov Adam optimizer [14] were used to optimize the weight parameters.

Supplemental Tables

Table I. Evaluation and acquisition of variables used in the model

**Patient demographics**	**Cardiological examinations**
Age (years)	#ECG parameters
Sex	**Echocardiogram parameters
**Baseline clinical variables**	††**Medication History**
Height (cm)	**Brain MRI findings**
Weight (kg)	‡‡Lateralization features
*Systolic blood pressure in ER (mmHg)	§§Lesion patterns on diffusion-weighted image
*Diastolic blood pressure in ER (mmHg)	||||SVS
*Heart rate in ER	||||Size of SVS
**Vascular risk factors**	##Intracranial and/or extracranial atherosclerosis
†Smoking status	***Hemorrhagic transformation
Congestive heart failure	**Laboratory examinations**
Hypertension	†††Complete blood count
Diabetes mellitus	†††Lipid profile
Coronary artery disease	Initial glucose (mg/dL)
Peripheral artery disease	Fasting glucose (mg/dL)
Prior history of stroke	HbA1c (%)
Prior history of transient ischemic attack	ESR (mm/h)
**Stroke details**	CRP (mg/dL)
Stroke onset date and time	CK-MB (ng/dL)
TOAST classification	Troponin I (ng/dL)
‡Recanalization therapy	Serum creatinine (mg/dL)
Initial NIHSS	§§§Proteinuria
Initial mRS	D-dimer (ug/mL)
END	FDP (ug/mL)
**AF details**	Fibrinogen (mg/dL)
§AF type	Homocysteine (μmol/L)
||AF time of diagnosis	||||||Liver function test panel
$CHADS_2$ score	
CHA_2DS_2-VASc score	

Abbreviations; ER, emergency room; TOAST, Trial of Org 10172 in Acute Stroke Treatment [15]; NIHSS, National Institutes of Health Stroke Scale; mRS, modified Rankin scale; END, early neurological deterioration; AF, atrial fibrillation; ECG, electrocardiography; $CHADS_2$ score, stroke risk stratification schemes for AF patients [16] (score assigns 1 point for congestive heart failure, hypertension, diabetes mellitus and 2 points for age ≥ 75 years, prior stroke or transient ischemic attack); CHA_2DS_2-VASc score, stroke risk stratification schemes for AF patients [17] (score assigns 1 point for congestive heart failure, hypertension, diabetes mellitus, vascular disease, age ≥ 65 years, female, and 2 points for age ≥ 75 years, prior stroke or transient ischemic attack); SVS, susceptibility vessel sign; ESR, erythrocyte sedimentation rate; CRP, C-reactive protein; CK-MB, creatine kinase-MB; FDP, fibrin degradation product

* The first blood pressure and heart rate when the patient arrived at the emergency room were measured

† Subdivided into pack-years, non-smoker, current smoker, ex-smoker who quit within the previous 5 years, and ex-smoker who quit more than 5 years previous

‡ Subdivided into none, intravenous recombinant tissue plasminogen activator therapy only, endovascular thrombectomy only, and both

§ Includes paroxysmal atrial fibrillation or persistent atrial fibrillation

|| Subdivided into diagnosis prior to admission, diagnosis during hospitalization, and diagnosis

after discharge
Includes ECG results (subdivided into normal sinus rhythm, AF with controlled heart rate, AF with rapid ventricular response, AF with slow ventricular response, and atrial flutter), PR interval, QRS duration, QT interval, QTc interval, P-axis, QRS, and T
** Includes wall motion abnormality, left atrial diameter, left ventricular ejection fraction, LA volume, and left ventricular and atrial thrombus
†† Includes medication history of aspirin, clopidogrel, dipyridamole, cilostazol, triflusal, ticlopidine, other antiplatelet agents, warfarin, apixaban, dabigatran, rivaroxaban, edoxaban, diuretics, calcium blockers, beta blockers, angiotensin-converting enzyme inhibitors, angiotensin II receptor blockers, alpha blockers, anti-diabetes mellitus medications, non-steroidal anti-inflammatory drugs and/or dose and type of statins including atorvastatin, rosuvastatin, simvastatin, or others
‡‡ Subdivided into right anterior circulation lesion, left anterior circulation lesion, right posterior circulation lesion, left posterior circulation lesion, bilateral or diffuse multifocal circulation lesions.
§§ Subdivided into single cortico-subcortical, cortical, subcortical (>15 mm), small scattered lesion in one vascular territory, confluent and an additional lesion in one vascular territory, and multiple lesions in multiple vascular territories [18]
|||| The presence and size of the susceptible vessel sign on gradient-echo images were measured [19]
Arterial steno-occlusion was defined as at least 50% narrowing or occlusion in intracranial and/or extracranial arteries using the NASCET method. It was subdivided into symptomatic, asymptomatic, and non-symptomatic [20]
*** Hemorrhagic transformation was firstly classified into none vs hemorrhagic transformation type 1 vs hemorrhagic transformation type 2 vs parenchymal hematoma type 1 vs parenchymal hematoma type 2 vs symptomatic intracranial hemorrhage.[1] Then subdivided into none *vs*. non-significant hemorrhagic transformation (including hemorrhagic infarction type 1, 2, and parenchymal hematoma type 1) vs significant hemorrhagic transformation(including parenchymal hematoma type 2 and symptomatic intracranial hemorrhage) [2].
††† Includes hemoglobin (mg/dL), hematocrit (%), white blood cell count (10^3/μL), and platelet count (10^3/μL)
‡‡‡ Includes total cholesterol (mg/dL), triglycerides (mg/dL), high-density lipoprotein cholesterol (mg/dL), and low-density lipoprotein cholesterol (mg/dL)
§§§ Using the dipstick test, subdivided into none, 1+, 2+, 3+, or more
|||||| Includes total bilirubin (mg/dL), aspartate aminotransferase (AST, U/L), alanine aminotransferase (ALT, U/L), and alkaline phosphatase (ALP, U/L)

Table II. Comparison of baseline clinical characteristics

Variables	END (-) (n=2,045)	END (+) (n=318)	*P value*
Age, mean (SD)	73.5 (9.9)	75.1 (9.6)	0.005
Female (%)	987 (48.3%)	185 (58.2%)	< .001
HTN (%)	1,392 (68.1%)	239 (75.2%)	0.013
DM (%)	531 (26.0%)	84 (26.4%)	0.882
Dyslipidemia (%)	417 (20.4%)	66 (20.8%)	0.896
Previous stroke history (%)			0.008
None	1,448 (70.8%)	214 (67.4%)	
Ischemic	503 (24.6%)	85 (26.6%)	
Hemorrhagic	32 (1.6%)	14 (4.4%)	
Both	21 (1.0%)	2 (0.6%)	
Unknown	41 (2.0%)	3 (0.9%)	
AF time of diagnosis (%)			0.001
Known AF	1,015 (51.6%)	120 (40.3%)	
Diagnosis during admission	948 (48.2%)	178 (59.7%)	
Diagnosis after discharge	4 (0.2%)	0 (0%)	
$CHADS_2$ score	2.1 (1.3)	2.3 (1.4)	0.004
CHA_2DS_2-VASc score	3.4 (1.6)	3.7 (1.7)	< .001
Initial NIHSS score	9.6 (7.6)	13.2 (7.8)	< .001
Systolic BP at ER, mmHg	146 (28)	149 (30)	0.027
Diastolic BP at ER, mmHg	85 (15)	87 (16)	0.112
Laboratory findings			
Hemoglobin, g/dL	13.5 (1.9)	13.6 (2.0)	0.423
WBC, 10^3/μL	8.3 (3.2)	8.9 (3.6)	0.003
PLT, 10^3/μL	203.8 (74.2)	211.0 (73.8)	0.108
CRP, mg/dL	3.98 (15.18)	4.25 (18.68)	0.080
Initial glucose, mg/dL	138.5 (75.5)	145.7 (49.7)	0.002
Fasting glucose, mg/dL	121.7 (39.5)	131.9 (44.4)	< .001
HbA1c (%)	6.0 (1.5)	6.2 (1.3)	0.075
Proteinuria (%)			0.022
Negative	1,322 (78.3%)	201 (70.3%)	
1+	212 (12.6%)	46 (16.1%)	
2+	115 (6.8%)	28 (9.8%)	
3+ or more	39 (2.3%)	11 (3.8%)	
D-dimer, (μg/mL)	32.2 (127.7)	51.0 (175.5)	< .001
FDP, (μg/mL)	11.9 (32.3)	16.7 (40.9)	0.009
Fibrinogen, (mg/dL)	304.2 (129.5)	306.9 (111.7)	0.566
Homocysteine, (μmol/L)	13.9 (50.2)	12.6 (12.8)	0.524
Total cholesterol, mg/dL	162.6 (37.4)	167.6 (40.0)	0.034
TG, mg/dL	96.0 (62.1)	95.7 (55.1)	0.768
HDL, mg/dL	49.9 (23.4)	53.5 (24.1)	0.017
LDL, mg/dL	96.4 (34.2)	95.6 (38.1)	0.234
CK-MB, mg/dL	3.4 (6.0)	3.5 (4.2)	0.585
Troponin I, mg/dL	0.10 (0.52)	0.29 (1.76)	0.029

Categorical variables are presented as number (percentage), and continuous variables are presented as mean (standard deviation).

Owing to missing values, the total number of some variables are different. The number of missing values for each variable are listed in Supplemental Table IV.

Abbreviations: END, early neurological deterioration; HTN, hypertension; DM, diabetes mellitus; AF, atrial fibrillation; NIHSS, National Institutes of Health Stroke Scale; BP, blood pressure; ER, emergency room; WBC, white blood cell count; PLT, platelet count; CRP, C-reactive protein; FDP, fibrin degradation product; TG, triglycerides; HDL, high-density lipoprotein; LDL, low-density lipoprotein; CK-MB, creatine kinase MB fraction.

Table III. Comparison of MRI variables

Variables	No END	END	p-value
Lateralization, n=2142 (%)			< .001
Right anterior	679 (36.3%)	117 (43.3%)	
Left anterior	642 (34.3%)	81 (30.0%)	
Posterior	299 (16.0%)	18 (6.7%)	
Bilateral or diffuse multifocal	252 (13.5%)	54 (20.0%)	
DWI patterns, n=1975 (%)			< .001
Single cortico-subcortical	425 (24.8%)	83 (32.2%)	
Cortical	174 (10.1%)	11 (4.3%)	
Subcortical (>15 mm)	128 (7.5%)	12 (4.7%)	
Subcortical (<15 mm)	89 (5.2%)	8 (3.1%)	
Small scattered lesion in one vascular territory	197 (11.5%)	22 (8.5%)	
Confluent and an additional lesion in one vascular territory	389 (22.7%)	63 (24.4%)	
Multiple lesions in multiple vascular territories	315 (18.3%)	59 (22.9%)	
SVS, n=1900 (%)	412 (20.1%)	96 (30.1%)	< .001
SVS size, n=429 (Mean, cm)	1.23 (0.68)	1.28 (0.65)	0.353
Hemorrhagic transformation n = 2086 (%)	1825	261	< .001
None	1,512 (82.8%)	201 (77.0%)	
Non-significant hemorrhagic transformation	275 (15.1%)	30 (18%)	
Significant hemorrhagic transformation	38 (2.1%)	13 (5%)	
Intracranial atherosclerosis n=2134 (%)			< .001
No steno-occlusion	873 (46.8%)	91 (33.7%)	
Steno-occlusion	991 (53.2%)	179 (66.3%)	
Extracranial atherosclerosis n=2125(%)			0.015
No steno-occlusion	1420 (76.5%)	186 (69.4%)	
Steno-occlusion	437 (23.5%)	82 (30.6%)	

Abbreviations: DWI, diffusion-weighted image; SVS, susceptible vessel sign.

Table IV. Missing values

Variables	Variable type	Missing values N (%)
**Clinical characteristics**		
**Height**	Continuous	97 (4.1%)
**Weight**	Continuous	14 (0.6%)
**Heart rate in ER**	Continuous	1 (0.0004%)
**Initial NIHSS score**	Continuous	5 (0.2%)
**Initial mRS score**	Continuous	629 (26.6%)
**Time of AF diagnosis**	Categorial	98 (4.1%)
**ECG Results**	Categorial	84 (3.6%)
**ECG QRS**	Continuous	368 (15.6%)
**ECG QT**	Continuous	368 (15.6%)
**ECG QTc**	Continuous	375 (15.9%)
**ECG QRS-axis**	Continuous	1106 (46.8%)
**ECG T-axis**	Continuous	1119 (47.4%)
**LA diameter**	Continuous	501 (21.2%)
**Medication history: dipyridamole**	Categorial	158 (6.7%)
**Medication history: other antiplatelets**	Categorial	63 (2.7%)
**Medication history: pioglitazone**	Categorial	57 (2.4%)
**Medication history: other DM drugs**	Categorial	18 (0.8%)
**Medication history: NSAIDs**	Categorial	7 (0.3%)
**MRI variables**		
**Lateralization**	Categorial	221 (9.4%)
**DWI patterns**	Categorial	388 (16.4%)
**SVS**	Categorial	463 (19.6%)
**Hemorrhagic transformation**	Categorial	277 (11.7%)
**Intracranial atherosclerosis**	Categorial	229 (9.7%)
**Extracranial atherosclerosis**	Categorial	238 (10%)
**Laboratory variables**		
**Hemoglobin**	Continuous	13 (0.6%)
**Hematocrit**	Continuous	61 (2.6%)

**WBC**	Continuous	12 (0.5%)
**PLT**	Continuous	12 (0.5%)
**ESR**	Continuous	737 (31.2%)
**CRP**	Continuous	200 (8.5%)
**Initial glucose**	Continuous	109 (4.6%)
**Fasting glucose**	Continuous	276 (11.7%)
**$HbA1_C$**	Continuous	239 (10.1%)
**Uric acid**	Continuous	447 (18.9%)
**Serum creatinine**	Continuous	43 (1.8%)
**Proteinuria**	Categorial	392 (16.6%)
**PT**	Continuous	52 (2.2%)
**aPTT**	Continuous	238 (10.1%)
**D-dimer**	Continuous	490 (20.7%)
**FDP**	Continuous	1141 (48.3%)
**Fibrinogen**	Continuous	444 (18.8%)
**Homocysteine**	Continuous	462 (19.6%)
**Total cholesterol**	Continuous	43 (1.8%)
**Triglycerides**	Continuous	170 (7.2%)
**HDL**	Continuous	169 (7.2%)
**LDL**	Continuous	166 (7%)
**AST**	Continuous	44 (1.9%)
**ALT**	Continuous	44 (1.9%)
**ALP**	Continuous	289 (12.2%)
**Total bilirubin**	Continuous	239 (10.1%)
**CK-MB**	Continuous	640 (27.1%)
**Troponin I**	Continuous	1186 (50.2%)

Abbreviations: ER, emergency department; NIHSS, National Institutes of Health Stroke Scale; mRS, modified Rankin scale; AF, atrial fibrillation; ECG, electrocardiogram; LA, left atrium; DM, diabetes mellitus; NSAID, non-steroidal anti-inflammatory drug; DWI, diffusion-weighted image; SVS, susceptible vessel sign size; WBC, white blood cell count; PLT, platelet count; ESR, erythrocyte sedimentation rate; CRP, C-reactive protein; PT, Prothrombin time; aPTT, activated partial thromboplastin time; FDP, fibrin degradation product; HDL, high-density lipoprotein; LDL, low-density lipoprotein; AST, aspartate aminotransferase; ALT, alanine aminotransferase; ALP, alkaline phosphatase; CK-MB, creatine kinase MB fraction.

Supplemental Figures

Figure I. Flowchart of data preprocessing and machine learning model construction

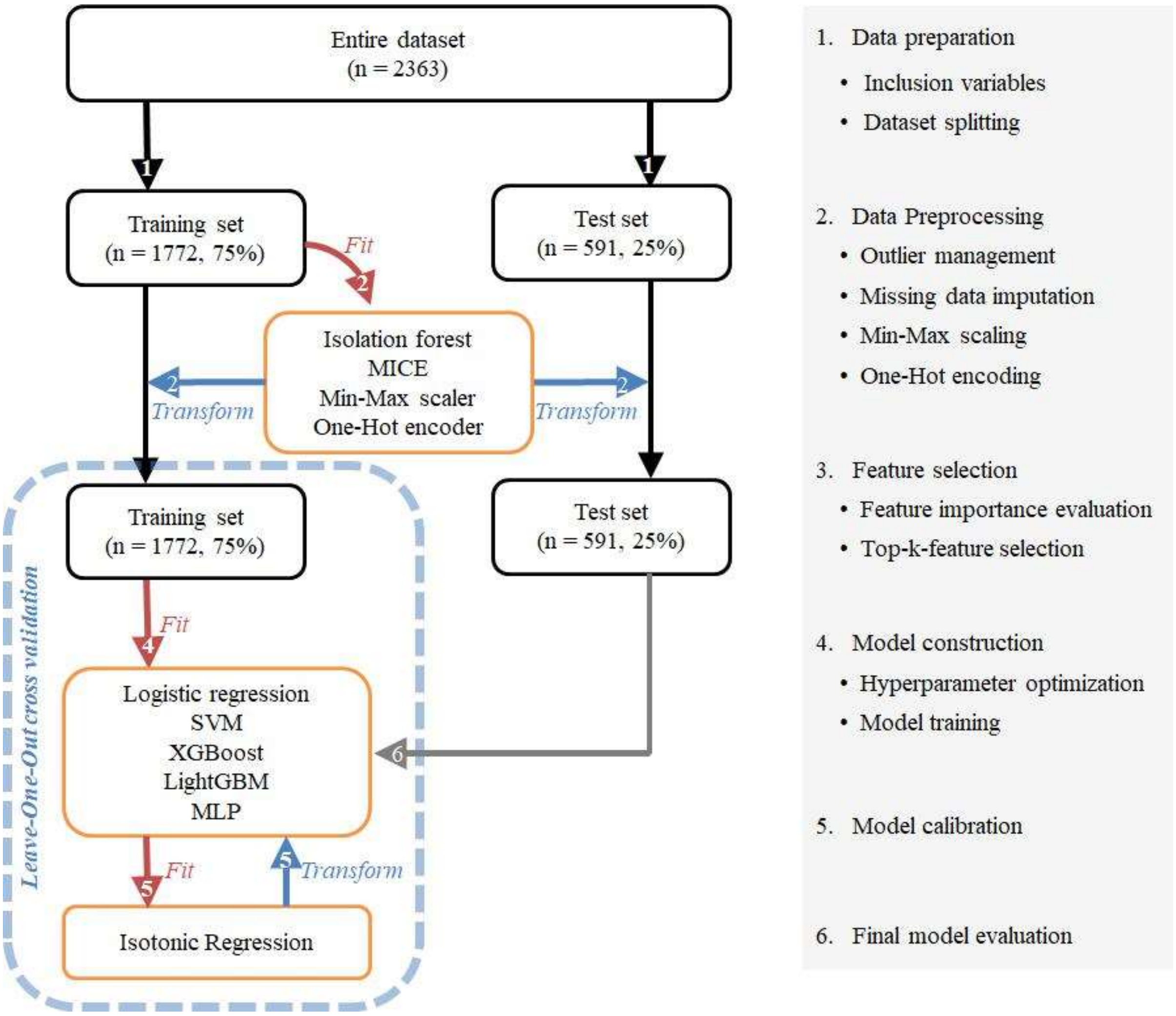

Abbreviations: MICE, Multiple imputation by chained equations; SVM, Support vector machine; XGBoost, Extreme gradient Boosting; LightGBM, Light gradient boosting machine; MLP, Multilayer perceptron

Figure II. SHAP value changes according to feature

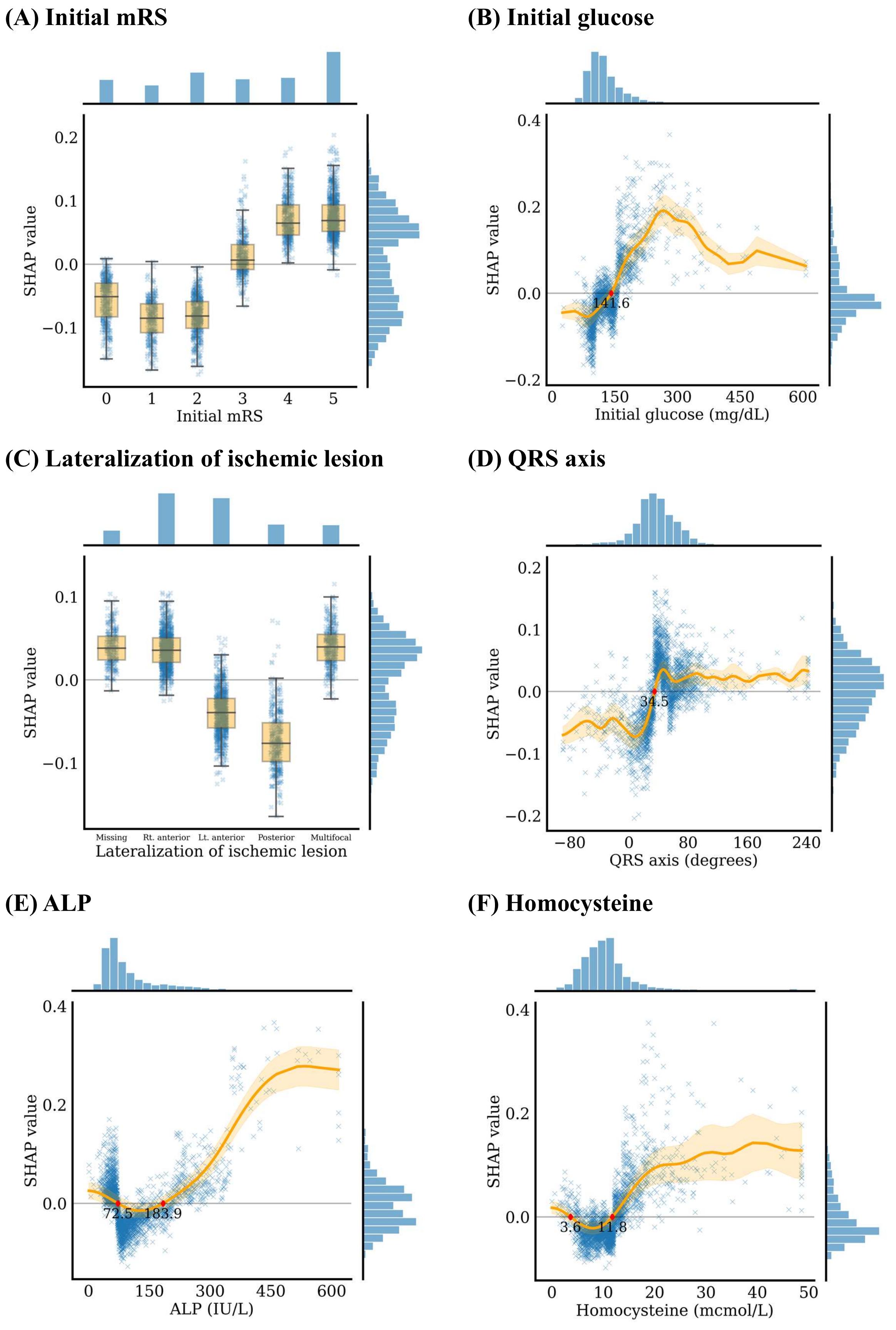

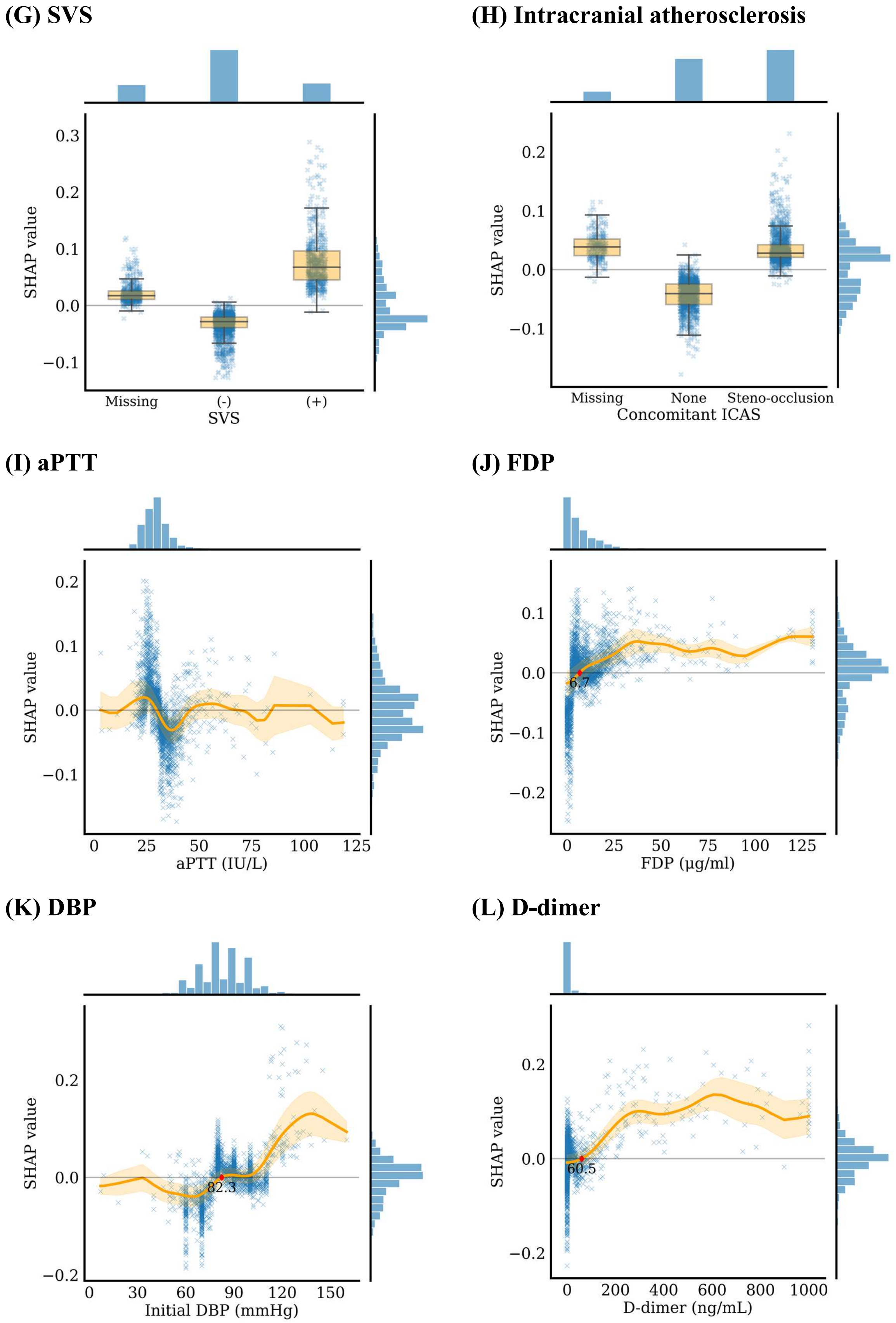
(G) SVS
SHAP value
Missing
(-)
(+)
SVS
(H) Intracranial atherosclerosis
SHAP value
Missing
None
Steno-occlusion
Concomitant ICAS
(I) aPTT
SHAP value
aPTT (IU/L)
(J) FDP
SHAP value
6.7
FDP (μg/ml)
(K) DBP
SHAP value
82.3
Initial DBP (mmHg)
(L) D-dimer
SHAP value
60.5
D-dimer (ng/mL)

(M) Hemorrhagic transformation

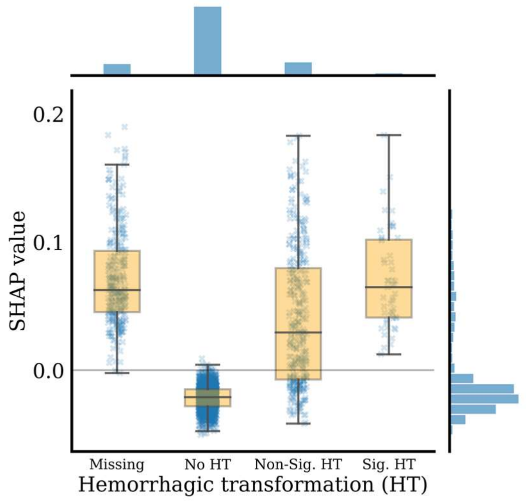

(N) AST

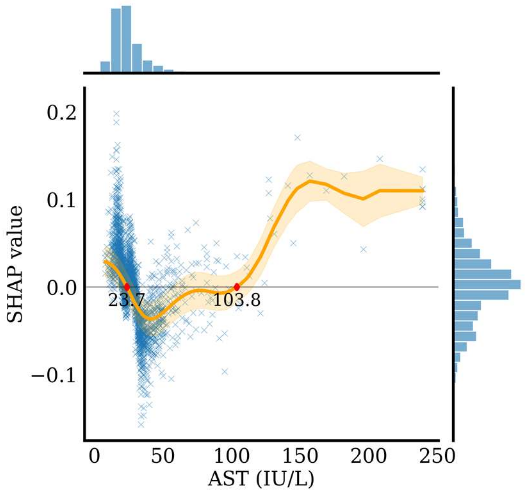

(O) Hematocrit

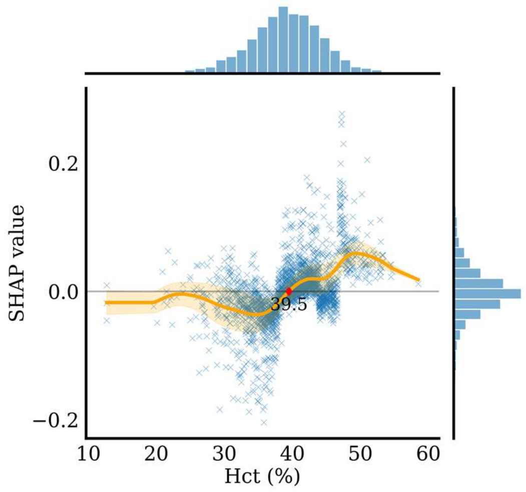

(P) Uric acid

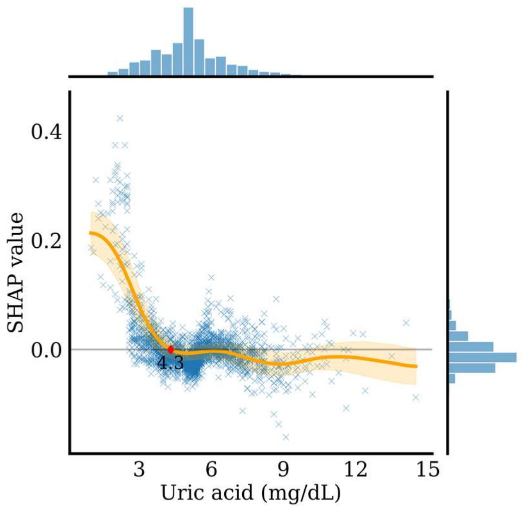

(Q) Total cholesterol

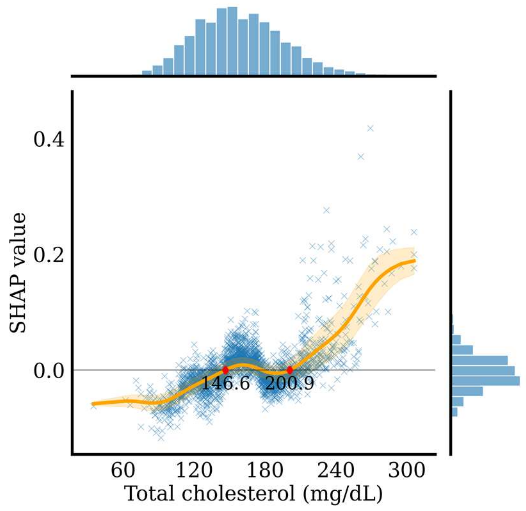

(R) T axis

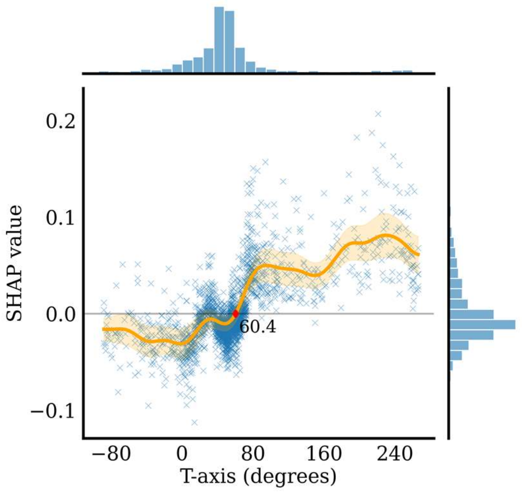

(S) Total bilirubin

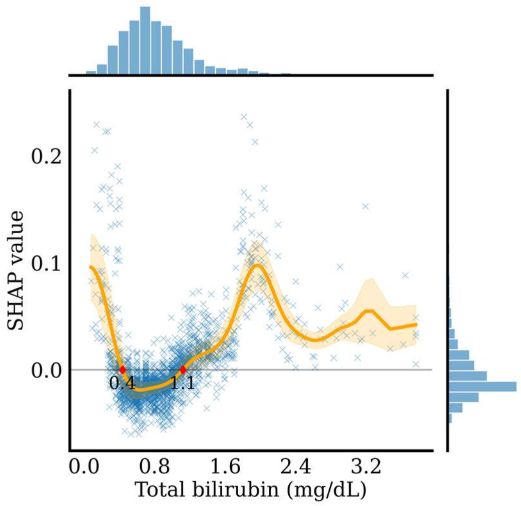

Abbreviations: mRS, modified Rankin scale; ALP, alkaline Phosphatase; SVS, susceptible vessel sign; aPTT, activated partial thromboplastin time; FDP, fibrin degradation product; DBP Diastolic blood pressure; HT, Hemorrhagic transformation; none-sig. HT, none-significant hemorrhagic transformation; Sig. HT, Significant hemorrhagic transformation; AST, aspartate aminotransferase; Hct, Hematocrit.

Figure III. Representative cases with SHAP values

(A)

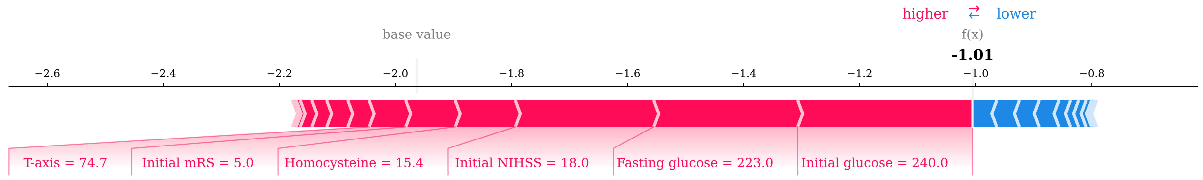

(B)

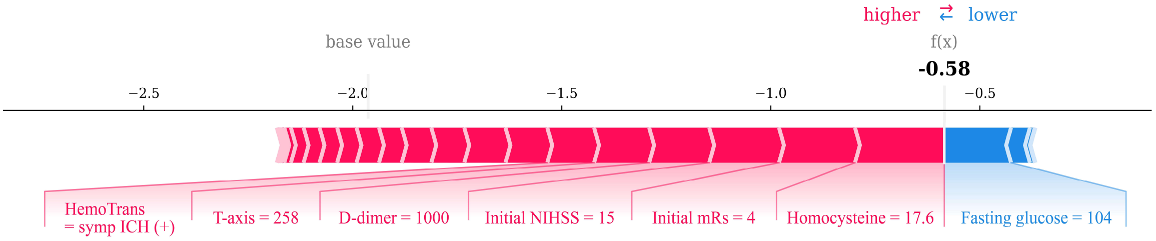

(C)

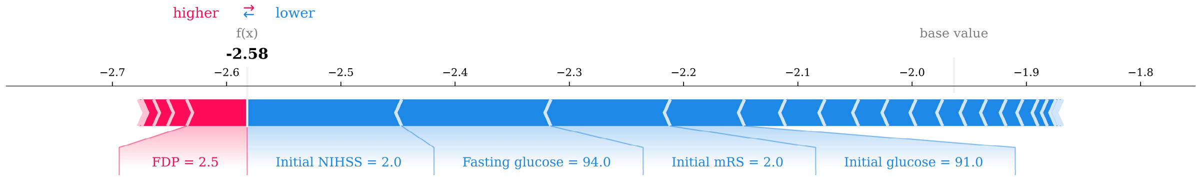

(D)

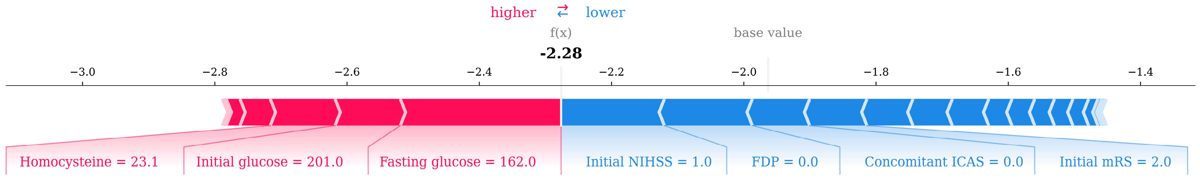

Red indicates a positive contribution to END prediction, and blue indicates a negative contribution. The area of each bar represents the degree of contribution to predictive power. Case (A) represents a typical case where glucose and NIHSS score, the most important contributing factors, contributed the most to END prediction. In case of (B), even though low fasting glucose contributed negatively to END, END was positively predicted since homocysteine, initial mRS score, initial NIHSS score and d-dimer had a more positive

contribution. Case (C) shows negative prediction of END because most influential factors (initial NIHSS score and fasting glucose level) had a negative contribution in the model, even though intracranial atherosclerosis was present. In the case of (D), a high fasting glucose level positively contributed to the model, and a low initial NIHSS score negatively contributed to the model. Considering both features, the net contribution was close to zero. However, other features, including FDP, concomitant intracranial atherosclerosis, and initial mRS score, negatively contributed more to the model, making it a negative prediction of END.

Supplemental References

1. Larrue V, von Kummer RR, Müller A, Bluhmki E. Risk factors for severe hemorrhagic transformation in ischemic stroke patients treated with recombinant tissue plasminogen activator: A secondary analysis of the european-australasian acute stroke study (ECASS II). *Stroke* **32**, 438–441 (2001).
2. Berger, et al. "Hemorrhagic transformation of ischemic brain tissue: asymptomatic or symptomatic?." *Stroke* **32** 1330-1335 (2001).
3. Liu FT, Ting KM, Zhou Z-H. Isolation-based anomaly detection. *ACM Trans Knowl Discov Data.* **6**, 1–39 (2012).
4. van Buuren S, Groothuis-Oudshoorn K. Mice: Multivariate imputation by chained equations in r. *J Stat Softw.* 1–68 (2010).
5. Gregorutti B, Michel B, Saint-Pierre P. Correlation and variable importance in random forests. *Stat Comput.* **27**, 659–678 (2017).
6. Fan R-E, Chang K-W, Hsieh C-J, Wang X-R, Lin C-J. Liblinear: A library for large linear classification. *JMLR.* **9**, 1871–1874 (2008).
7. Chen, T., & Guestrin, C. Xgboost: A scalable tree boosting system. Paper presented at: Proceedings of the 22nd acm sigkdd international conference on knowledge discovery and data mining 785-794 (2016).
8. Chang C-C, Lin C-J. Libsvm: A library for support vector machines. *ACM Trans Intel Syst Technol.***2**, 1–27 (2011).
9. Ke G, et al. LightGBM: A highly efficient gradient boosting decision tree. *Advances in Neural Information Processing Systems*. 3146–3154 (2017).
10. LeCun Y, Bengio Y, Hinton G. Deep learning. *Nature.* **521**, 436–444 (2015).
11. Ioffe S, Szegedy C. Batch normalization: Accelerating deep network training by reducing internal covariate shift. *International conference on machine learning*, PMLR **37** 448-456 (2015).
12. Srivastava N, Hinton G, Krizhevsky A, Sutskever I, Salakhutdinov R. Dropout: A simple way to prevent neural networks from overfitting. *JMLR.* **15**, 1929–1958 (2014).
13. Hanin B, Rolnick D. How to start training: The effect of initialization and architecture. *Advances in Neural Information Processing Systems*. **571–581 (**2018).
14. Dozat T. Incorporating nesterov momentum into adam. *ICLR. (*2016).
15. Adams Jr, et al. "Classification of subtype of acute ischemic stroke. Definitions for use in a multicenter clinical trial. TOAST. Trial of Org 10172 in Acute Stroke Treatment." S*troke* **24,** 35-41 (1993).
16. Gage BF, et al. Validation of clinical classification schemes for predicting stroke: Results from

the national registry of atrial fibrillation. *JAMA.* **285**, 2864–2870 (2001).

17. Lip GYH, Nieuwlaat R, Pisters R, Lane DA, Crijns HJGM. Refining clinical risk stratification for predicting stroke and thromboembolism in atrial fibrillation using a novel risk factor-based approach: The euro heart survey on atrial fibrillation. *Chest.***137**, 263–272 (2010).
18. Kim BJ, et al. Imaging characteristics of ischemic strokes related to patent foramen ovale. *Stroke.***44**, 3350–3356 (2013).
19. Naggara O, et al. T2*"susceptibility vessel sign" demonstrates clot location and length in acute ischemic stroke. *PLoS One.* **8**, e76727 (2013).
20. Moneta GL, et al. Correlation of north american symptomatic carotid endarterectomy trial (NASCET) angiographic definition of 70% to 99% internal carotid artery stenosis with duplex scanning. *J Vasc Surg.***17**, 152–159 (1993).
